# Prospective evaluation of intravitreal bevacizumab for ischemic central retinal vein occlusion

**DOI:** 10.1186/s40942-019-0183-x

**Published:** 2019-07-26

**Authors:** Leangelo Hall, Luma Paiva Frizzera, Laura Fernandes Coelho, Pedro Carlos Carricondo, Maria Kiyoko Oyamada, Sergio Luis Gianotti Pimentel, Maria Fernanda Abalem

**Affiliations:** 1 Harvard Medical School, Boston, MA USA; 2 Department of Ophthalmology and Otolaryngology, University of Sao Paulo Medical School, São Paulo, Sao Paulo Brazil; 3 Department of Ophthalmology and Visual Sciences, W. K. Kellogg Eye Center, University of Michigan, 1000 Wall Street, Ann Arbor, MI 48150 USA

**Keywords:** Ischemic central retinal vein occlusion, Bevacizumab, Macular edema, Choroidal thickness

## Abstract

**Background:**

Although previous studies have evaluated the effect of anti-VEGF therapies for central retinal vein occlusion (CRVO) patients, the majority of previous studies have excluded or included a very small number of patients with ischemic CRVO (iCRVO). The aim of our study is to examine the effects of bevacizumab on macular edema secondary to ischemic central retinal vein occlusion, as well as the effects on central choroidal thickness and best-corrected visual acuity.

**Methods:**

In this prospective, interventional case series, iCRVO was defined by the presence of ≥ 10 or more disc diameter areas of retinal nonperfusion by fluorescein angiography (FA) and by the presence of a b/a ratio less than 1.5 by full-field electroretinogram (ffERG). Nine eyes with iCRVO received monthly bevacizumab 0.5 mg injections at baseline and months 1 to 5 for a maximum of six injections. Main outcome measures were visual acuity (Snellen), central foveal thickness, and central choroidal thickness as measured by Spectral-Domain Optical Coherence Tomography (SD-OCT) at baseline and at 6 month following initial intravitreal bevacizumab injection. Pairwise t-tests and the Wilcoxon signed-rank test were conducted to compare the outcome measures.

**Results:**

After intravitreal administration of bevacizumab, there was a significant reduction of central foveal thickness from 858 ± 311 μm at baseline to 243 ± 106 μm at the 6-month follow-up, as well as a significant reduction of central choroidal thickness from 282 ± 38 μm at baseline to 227 ± 56 μm at the 6-month follow-up (p = 0.0006, p = 0.0003 respectively). The visual acuity worsened from a median of 1.3 to 1.7 (p = 0.02).

**Conclusion:**

In patients with iCRVO, intravitreal bevacizumab led to a reduction of central macular edema and central choroidal thickness, but a worsening of visual acuity. Intravitreal bevacizumab reduces macular edema but is not able to overcome the poor prognosis of iCRVO.

## Introduction

Retinal vein occlusion (RVO) is the second most common blinding vascular disorder of the retina [1]. Previous studies have demonstrated that ischemic CRVO (iCRVO) confers a poorer prognosis than non-ischemic CRVO (non-iCRVO) [2, 3]. For example, patients with iCRVO have significantly higher levels of aqueous and vitreous vascular endothelial growth factor (VEGF) concentrations as compared to patients with non-iCRVO [4, 5]. The higher levels of VEGF in iCRVO play a role in development of neovascularization, which increases the risk for vision-threatening complications, such as vitreous hemorrhage, neovascular glaucoma, and tractional retinal detachment [2, 6]. Even after sequalae secondary to CRVO resolve, iCRVO patients fare more poorly compared to non-iCRVO patients [2].

Intravitreal anti-VEGF drugs have become first-line therapy for patients with macular edema secondary to CRVO [7]. Many studies have successfully demonstrated the efficacy of anti-VEGF drugs in CRVO [8–14]. For example, the CRUISE randomized controlled trial demonstrated that CRVO patients who received intravitreal ranibizumab injections had a significant reduction of central foveal thickness at 6-months as compared to the control group [8]. Similarly, Epstein et al. [9] conducted a randomized controlled trial that showed that patients who received intravitreal bevacizumab injections had best-corrected visual acuity BCVA that improved by 14.1 ETDRS letters at 24 weeks compared with a decrease of 2.0 letters in the control group.

While previous studies have shed light on the effect of anti-VEGF drugs for CRVO patients, the majority of previous studies have excluded or included a very small number of patients with iCRVO. For example, GALILEO found that aflibercept reduced macular edema secondary to CRVO, however, only 14 of the 177 patients included in the study had iCRVO and separate analysis for outcomes of iCRVO patients was not conducted given the small number of patients [13]. In addition, CRYSTAL was a prospective study that showed that intravitreal ranibizumab injections for CRVO patients led to significant improvement of visual acuity, with 63.8% of patients gaining ≥ 10 ETDRS (Early Treatment Diabetic Retinopathy Study) letters [14]. While 54 eyes had iCRVO in the CRYSTAL study, the definition was not given and the data specific to iCRVO and non-iCRVO patients was not provided separately, making it difficult to draw conclusions specific to iCRVO patients [14]. In our prospective case series, we aim to describe the effect of intravitreal bevacizumab on macular edema secondary to CRVO, as well as central choroidal thickness and best corrected visual acuity.

## Methods

### Study design

The current study was designed as a prospective case series and performed at the Ophthalmology Department at the University of São Paulo Medical School during 2012. The study was approved by the institutional review board and adhered to the tenets of the Declaration of Helsinki. The inclusion criteria were as follows: (1) Diagnosis of ischemic CRVO by an ophthalmologist. Ischemia was defined by the presence of ≥ 10 or more disc diameter areas of retinal nonperfusion by fluorescein angiography (FA) and by the presence of a b/a ratio less than 1.5 by full-field electroretinogram (ffERG). (2) Age of 18 years or older. (3) Diagnosis of iCRVO up to 6 months prior to study enrollment. (4) Baseline Snellen visual acuity worse than 20/200. Patients were excluded if they had presence of refractive error exceeding ± 6.00 diopters (D) of spherical equivalent, presence of more than 2 diopters of keratometric astigmatism, axial length of more than 26.5 mm, important media opacity resulting in poor OCT image quality, prior history of CRVO, history of any retinal disease, including diabetic retinopathy, uveitis, glaucoma or any other optic neuropathy, intraocular surgery in the past 3 months (including cataract surgery), intravitreal injections (steroids and/or anti-vascular endothelial growth factor) and/or laser treatment.

### Study protocol

All patients underwent a comprehensive ocular examination, including visual acuity on Snellen chart, biomicroscopy of anterior and posterior segments, intraocular pressure, and Spectral-Domain Optical Coherence Tomography (SD-OCT) (Spectralis; Heidelberg Engineering, Heidelberg, Germany) and ffERG. The ffERG was performed compliant with the standards of the International Society for Clinical Electrophysiology of Vision (ISCEV). All procedures were performed at standardized visits at baseline, at 1, 2, 3, 4, 5 and 6 months, before each bevacizumab injection, with a time window of ± 2 weeks. FA and ffERG were performed only at baseline as part of screening procedures.

Spectral-Domain Optical Coherence Tomography images were acquired using the automated eye alignment eye-tracking software (TruTrack; Heidelberg Engineering) to obtain 25 high-speed horizontal line scans of the macula of both eyes within the central 20° centered in the fovea. The central foveal thickness derived from the Early Treatment Diabetic Retinopathy Study (ETDRS) grid was selected as the outcome measure for evaluating the central macular edema (CME). SD-OCT with an EDI (enhanced depth imaging) protocol (horizontal and vertical scans, 20 × 20, 49 sections, high resolution mode, 30 frames) was also performed to obtain the central choroidal thickness (CCT). CCT was measured at the subfoveal position. The CCT measures were performed manually using the caliper of the Eye Explorer Software (v. 6.0.9.0; Heidelberg Engineering), with 80% zoom. The measure was obtained perpendicularly, from the outer edge of the hyperreflective retinal pigmented epithelium (RPE) to the hyperreflective inner sclera. All SD-OCT images were routinely obtained at the same time, avoiding diurnal variations of the choroidal and retinal thicknesses.

### Injection protocol

Patients received their monthly bevacizumab 0.5 mg injection at baseline and months 1 to 5 for a maximum of six injections. Sterile protocol for intravitreal injection included the use of 5% povidone–iodine solution, topical anesthesia, eyelid-speculum application, and intravitreal injection of 0.5 mg of bevacizumab via pars plana at the inferotemporal quadrant at 4 mm from the limbus in phakic eyes and 3.5 mm in pseudophakic eyes, followed by postoperative topical antibiotic eye drops.

### Statistical analysis

All data collection and analyses were performed using MS-Excel 2018 (Microsoft Corporation, USA). Continuous variables are presented as mean ± standard deviation or median (range). The primary outcome measures were: visual acuity, central foveal thickness and central choroidal thickness. Pairwise t-tests and the Wilcoxon signed-rank test were conducted to determine compare the outcome measures were statistically different between baseline and after treatment. p < 0.05 was considered significant.

## Results

A total of nine individuals (five females) with iCRVO were enrolled in the study. Five patients identified as white (55.6%), 2 as hispanic (22.2%), and 2 as black (22.2%). Eight patients had hypertension (88.9%) and 1 patient had diabetes mellitus (1.1%). The age of the patients ranged from 44 to 78 years (mean, 57.9 ± 10.6 years). Duration of symptoms before treatment ranged from 1 to 6 months (mean, 2.4 ± 1.6 months). All individuals completed the 6-month follow-up period and received all six intravitreal injections of bevacizumab. No patients experienced adverse events (endophthalmitis, retinal detachment, lens trauma) due to injections during the study, however, one patient had a vitreous hemorrhage and another patient developed neovascular glaucoma during the study period and despite intravitreal injections of bevacizumab. Table 1 shows the demographics and clinical characteristics of patients included in the study.

**Table 1:** Demographic and clinical characteristics

Eye no.	Gender	Ethnicity	Age, years	Duration of symptoms, months	Visual acuity baseline, logMAR	Visual acuity at 6 months, logMAR	CFT baseline, μm	CFT at 6 months, μm	CCT baseline, μm	CCT at 6 months, μm
1	Male	White	50	2	1.3	HM	839	241	235	173
2	Female	Black	58	1	1.7	1.7	1128	414	359	276
3	Male	White	64	2	1.3	1.7	317	400	251	177
4	Female	White	78	6	HM	NLP	1033	235	235	164
5	Male	Hispanic	48	2	1.3	1.7	726	242	302	188
6	Female	White	63	4	1.3	1.7	527	163	330	306
7	Male	Hispanic	44	1	1.7	1.7	1136	150	294	212
8	Female	Black	52	3	1.3	1.7	1261	240	268	256
9	Female	White	64	1	1	1.3	759	99	341	295

*HM* hand movements, *NLP* no light perception, *CFT* central foveal thickness, *CCT* central choroidal thickness

After intravitreal administration of bevacizumab, there was a significant decrease in visual acuity from a median of 1.3 to 1.7 (p = 0.02). Prior to study treatment the visual acuity of eyes was: 1 eye with 20/200, 5 eyes with 20/400, 2 eyes with 20/1000, and 1 eye with hand movements. At the 6-month follow-up the visual acuity of eyes was: 1 eye with 20/400, 6 eyes with 20/1000, 1 eye with hand movements, and 1 eye with no light perception. For the purposes of our study, visual acuity of hand movements was defined as 20/4000.

**Fig. 1:**
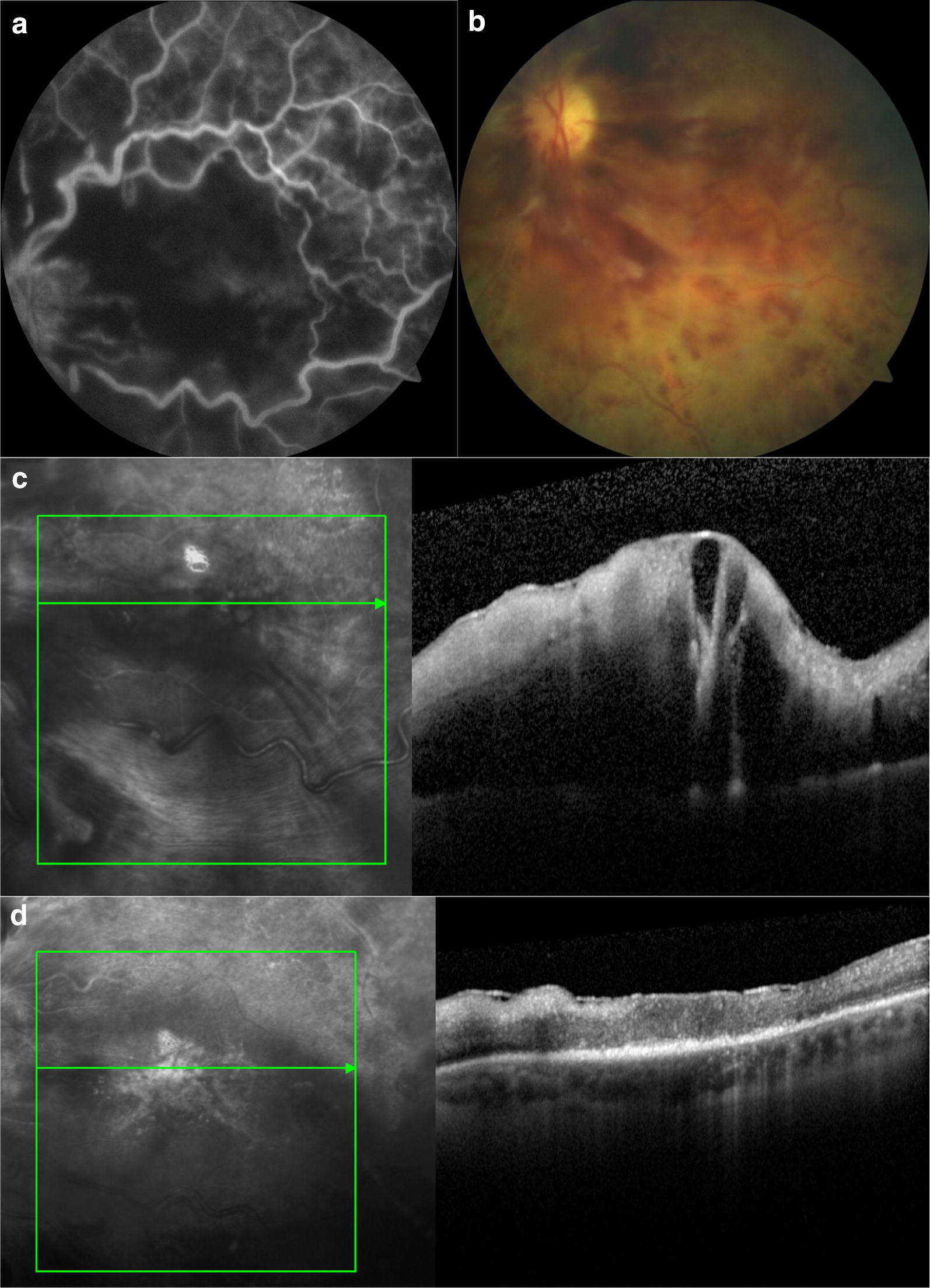
Change in retinal thickness after intravitreal bevacizumab. A 78-year-old patient presented with ischemic central retinal vein occlusion, which is shown by fluorescein angiography (**a**, **b**). He had symptoms for 6 months. Baseline visual acuity was reduced to hand movements. Optical coherence tomography shows baseline (**c**) and at the 6-month follow-up shows macular edema has decreased, the foveal contour has been lost due to the presence of the epiretinal membrane, and there is degeneration of the outer retinal layers. Although the macular edema resolves, the final acuity was no light perception

**Fig. 2:**
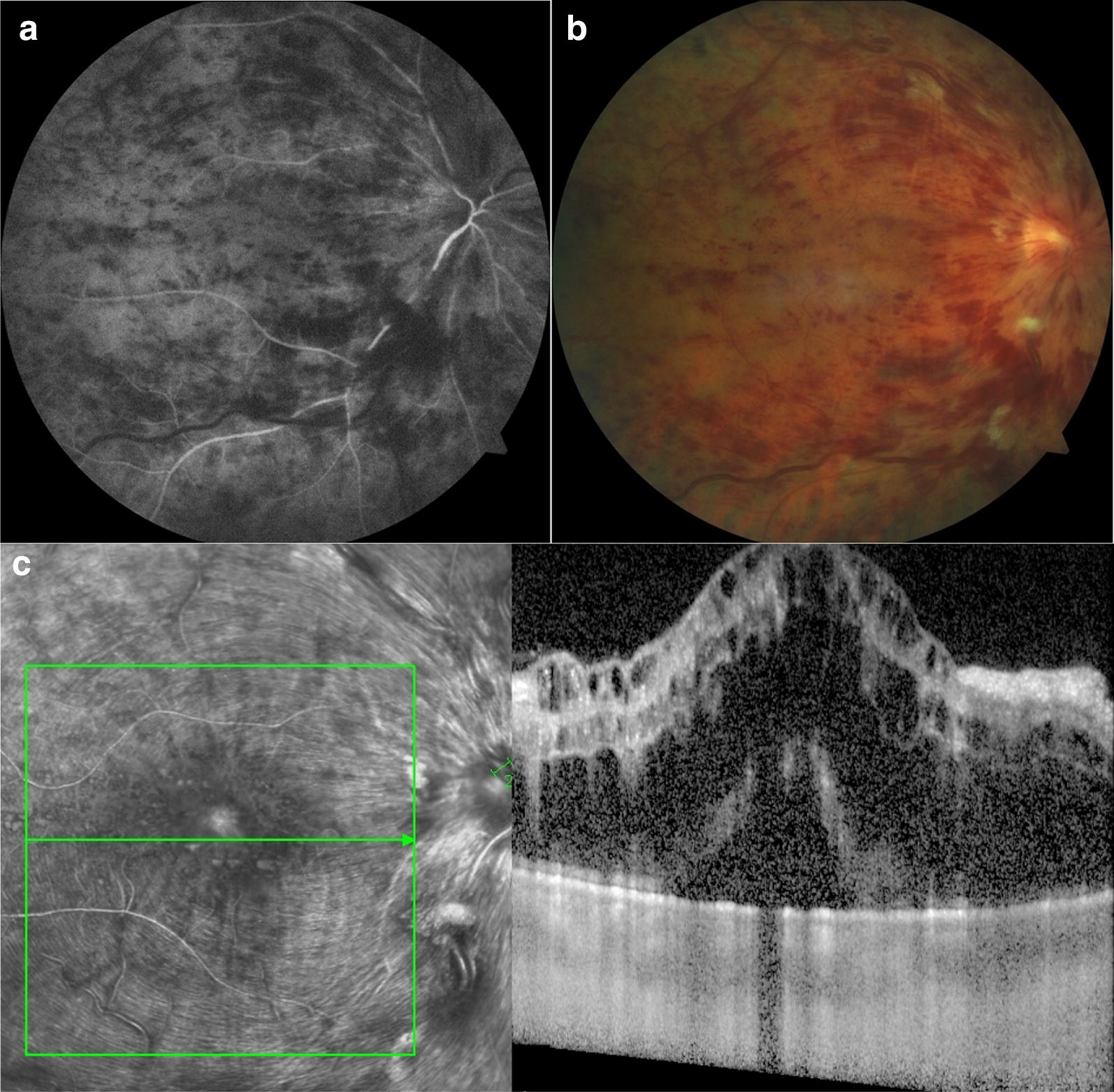
Change in retinal thickness after intravitreal bevacizumab. A 48-year-old patient presented with intraretinal hemorrhages in the four quadrants ischemic, macular edema, vascular tortuosity compatible with central retinal vein occlusion, which is shown by fluorescein angiography (**a**, **b**). He had symptoms for 2 months. Baseline visual acuity was reduced to 20/200, and the baseline optical coherence tomography shows macular edema and serous retinal detachment (**c**). At the 6-month follow-up macular edema has decreased, the foveal contour has been recovered, and there is degeneration of the outer retinal layers

Following study treatment, all patients had a significant decrease in both the central foveal thickness and central choroidal thickness. The mean central foveal thickness was 858 ± 311 μm at baseline and decreased to 243 ± 106 μm at the 6-month follow-up (p = 0.0006). The mean central choroidal thickness was 282 ± 38 μm at baseline and decreased 227 ± 56 μm at the 6-month time point (p = 0.0003). Figures 1 and 2 demonstrate the reduction of macular edema for two patients examined during the study (Table 2).

**Table 2:** Results for nine cases of ischemic central retinal vein occlusion

Finding	Baseline	6 months	p value
Visual acuity, logMAR	1.3 (1 to HM)	1.7 (1.3 to NLP)	0.02*
Central foveal thickness	858 ± 311	243 ± 106	0.0006^#^
Central choroidal thickness	282 ± 38	227 ± 56	0.0003^#^

Data are presented as median (range) or mean ± standard deviation

*HM* hand movements, *NLP* no light perception

*Wilcoxon signed-rank test

^#^Pairwise t-test

## Discussion

In our prospective case series, we evaluated the effect of intravitreal injections of bevacizumab on structural and functional outcome measures of patients with iCRVO. The results demonstrated after six consecutive bevacizumab injections over a 6-month period, patients had a significant decrease in central foveal thickness and central choroid thickness, however, significantly worse visual acuity. Patients did not have any drug-related complications during the study period.

Similar to our findings, previous studies have found that intravitreal injections of bevacizumab reduce central macular edema for CRVO patients [9, 12, 15–17]. There are previous studies that have specifically examined the effect of anti-VEGF drugs in iCRVO patients. For example, the COPERNICUS randomized control trial demonstrated that at a 6-month follow-up, 51.4% of 29 iCRVO eyes that received aflibercept gained > 15 letters as compared to 4.3% in the sham group [11]. The RAVE trial showed that iCRVO patients who received intravitreal ranibizumab had a mean visual acuity gain of 21.4 ETDRS letters at 36-months [11]. With the exception of Priglinger et al. [12] and Costa et al. [17], a major weakness of previous studies is that they did not delineated between iCRVO and non-iCRVO patients. Prigliner et al. [12] conducted a prospective, consecutive, noncomparative case series in which patients received an intravitreal injection of bevacizumab on day 1 and 4 weeks thereafter. Additional injections of bevacizumab were given within the 6-month study period if patients had not surpassed treatment success and did not meet criteria for treatment futility [12]. Treatment success was defined as BCVA of ≥ 79 ETDRS letters and average central retinal thickness by OCT as ≤ 225 μm [12]. If treatment had been stopped, it was reinitiated if retinal thickness increased by ≥ 50 μm or BCVA was < 74 ETDRS letters [12]. Treatment futility, defined as not achieving at least a decrease 50 μm of average central retinal thickness and an increase of BCVA of ≥ 5 letters [12]. The study found that the mean baseline of central retinal thickness significantly decreased from 535 ± 148 to 323 ± 116 for all CRVO patients, and from 534 ± 193 to 279 ± 127 for iCRVO patients [12]. Costa et al. [17] conducted a prospective study over 25 weeks in which ischemic central and hemicentral RVO patients received an intravitreal injection of bevacizumab at baseline every 12 weeks if macular edema recurrence was documented by OCT. The central macular thickness decreased from a baseline of 730.1 μm to 260.3 μm at the 25-week follow-up [17]. Table 3 demonstrates a comparison of the outcomes of our study with the previously described studies.

**Table 3:** Comparison between studies using bevacizumab for central retinal vein occlusion

Study	Type of CRVO	Eyes	Baseline visual acuity, logMAR	6 month visual acuity, logMAR	Baseline CFT, μm	6 month CFT, μm
Our study	iCRVO only	9	1.3 (1 to HM)	1.7 (1.3 to NLP)	858 ± 311	243 ± 106
Prigliner et al. [12]	iCRVO only	17	1.52 ± 0.34	1.06 ± 0.45	534 ± 193	279 ± 127
Costa et al. [17]	iCRVO and non-iCRVO	7	1.21 ± 0.36	0.68 ± 0.32	730 ± 257	260 ± 135

*CRVO* central retinal vein occlusion, *iCRVO* ischemic central retinal vein occlusion, *non-iCRVO* non-ischemic central retinal vein occlusion, *CFT* central foveal thickness

Our study also found that intravitreal injections of bevacizumab lead to a reduction of central choroidal thickness in iCRVO patients. Previous studies have seen a reduction of CCT in patients with diabetic retinopathy, and some postulate that this reduction is related to damage to the retina [18, 19]. The choroid is the primary source of oxygen and nutrition to the outer layers of the retina, and if the choroid is damaged from retinal tissue hypoxia or ischemia such as diabetes mellitus, this could result in decreased blood supply to the outer retina and thus damage to photoreceptors. It is possible that a similar phenomenon occurs with anti-VEGF injections. Increased choroidal thickness has also been found to be associated with better visual acuity [20]. It is difficult to draw conclusions about the reduction of CCT, however, future studies examining the effect of anti-VEGF agents could use CCT as an outcome measure given that the reduction of CCT has been found to be significantly correlated with reduction of macular edema [21, 22].

In contrast to the aforementioned studies, our study found no correlation between the structural and functional outcomes given that the visual acuity of patients significantly worsened. These findings could be explained by the poorer prognosis that iCRVO confers as compared to non-iCRVO [2]. Although this study was not comparative, an observational study of untreated patients showed that after resolution of macular edema, final visual acuity was reported to be 20/100 or better in 83% of non-iCRVO eyes as compared to 12% in iCRVO eyes [2]. Moreover, the damage to photoreceptors caused by CRVO may persist despite resolution of macular edema, and therefore the resolution of macular edema may not cause an improvement of visual acuity in these cases of severe macular ischemia [22]. Future studies using OCT at the study endpoint may shed light on residual retinal ischemia and correlation to final visual acuity. In the long-term the visual acuity of CRVO worsens, as shown by the HORIZON trial, which found that despite receiving intravitreal injections of ranibizumab for a year, the visual acuity CRVO eventually declined [23]. Furthermore, the natural history of CRVO is variable [2, 3]. Additionally, the Central Vein Occlusion Study found that the final visual acuity after CRVO is strongly linked to initial visual acuity, with only 19% of patients with initial visual acuity of worse than 20/200 having a final visual acuity of better than 20/200 [24]. Thus, an improvement for this group of patients is rare. Our study only included patients with visual acuity of 20/200 or worse, all of whom had a worsening of visual acuity or maintained their initial poor visual acuity.

Strengths of our study include: the prospective nature, strict criteria for defining iCRVO using fluorescein angiography and full-field electroretinogram, and a standardized protocol for imaging acquisition. In addition, patients did not receive any other form of treatment for CRVO, such as intravitreal corticosteroids. Finally, all patients received the same number of administrations of intravitreal bevacizumab every 4 weeks during a 6-month time period. Limitations of our study include the small sample size as well as the noncomparative nature of the study.

Our results suggest that patients that had received intravitreal bevacizumab treatment for iCRVO had reduction of central macular thickness and central choroidal thickness but worsening of visual acuity. Patients with iCRVO may not necessarily benefit from intravitreal bevacizumab injections as iCRVO confers a poorer prognosis as compared to non-iCRVO. Future studies are needed to ascertain the long-term effects of bevacizumab for iCRVO patients and to further examine the correlation between structural and functional outcomes.

## Conclusion

In patients with iCRVO, intravitreal bevacizumab led to a reduction of central macular edema and central choroidal thickness, but a worsening of visual acuity. Intravitreal bevacizumab reduces macular edema but is not able to overcome the poor prognosis of iCRVO.

## Data Availability

Additional data may be presented upon request.

## Abbreviations

BCVA: best-corrected visual acuity
CCT: central choroidal thickness
CFT: central foveal thickness
CME: central macular edema
CRVO: central retinal vein occlusion
ETDRS: Early Treatment Diabetic Retinopathy Study
FA: fluorescein angiography
ffERG: full-field electroretinogram
HM: hand movements
iCRVO: ischemic central retinal vein occlusion
NLP: no light perception
Non-iCRVO: non-ischemic central retinal vein occlusion
RPE: retinal pigmented epithelium
RVO: retinal vein occlusion
SD-OCT: Spectral-Domain Optical Coherence Tomography
VEGF: vascular endothelial growth factor

## References

[1] Ehlers J, Fekrat S (2011). Retinal vein occlusion: beyond the acute event. Surv Ophthalmol.

[2] Hayreh SS, Podhajsky PA, Zimmerman MB (2011). Natural history of visual outcome in central retinal vein occlusion. Ophthalmology.

[3] McIntosh RL, Rogers SL, Lim L, Cheung N, Wang JJ, Mitchell P (2010). Natural history of central retinal vein occlusion: an evidence-based systematic review. Ophthalmology.

[4] Noma H, Funatsu H, Mimura T, Eguchi S, Shimada K, Hori S (2011). Vitreous levels of pigment epithelium-derived factor and vascular endothelial growth factor in macular edema with central retinal vein occlusion. Curr Eye Res.

[5] Noma H, Funatsu H, Mimura T, Shimada K (2011). Influence of ischemia on visual function in patients with branch retinal vein occlusion and macular edema. Clin Ophthalmol.

[6] Hayreh SS, Zimmerman MB (2012). Ocular neovascularization associated with central and hemicentral retinal vein occlusion. Retina.

[7] Khayat M, Williams M, Lois N (2018). Ischemic retinal vein occlusion: characterizing the more severe spectrum of retinal vein occlusion. Surv Ophthalmol.

[8] Brown DM, Campochiaro PA, Singh RP, Li Z, Gray S, Saroj N (2010). Ranibizumab for macular edema following central retinal vein occlusion: six-month primary end point results of a phase III study. Ophthalmology.

[9] Epstein DLJ (2012). Bevacizumab for macular edema in central retinal vein occlusion: a prospective, randomized, double-masked clinical study. Ophthalmology.

[10] Brown DM, Heier JS, Clark WL, Boyer DS, Vitti R, Berliner AJ (2013). Intravitreal aflibercept injection for macular edema secondary to central retinal vein occlusion: 1-year results from the phase 3 COPERNICUS study. Am J Ophthalmol.

[11] Brown DM, Wykoff CC, Wong TP, Mariani AF, Croft DE, Schuetzle KL (2014). Ranibizumab in preproliferative (ischemic) central retinal vein occlusion: the rubeosis anti-VEGF (RAVE) trial. Retina.

[12] Priglinger SG, Wolf AH, Kreutzer TC, Kook D, Hofer A, Strauss RW (2007). Intravitreal bevacizumab injections for treatment of central retinal vein occlusion: six-month results of a prospective trial. Retina.

[13] Holz FG, Roider J, Ogura Y, Korobelnik J, Simader C, Groetzbach G (2013). VEGF trap-eye for macular oedema secondary to central retinal vein occlusion: 6-month results of the phase III GALILEO study. Br J Ophthalmol.

[14] Larsen M, Waldstein SM, Boscia F, Gerding H, Mone ´s J, Tadayoni R (2016). Individualized ranibizumab regimen driven by stabilization criteria for central retinal vein occlusion: twelve-MONTH results of the CRYSTAL study. Ophthalmology.

[15] Rosenfeld Philip J, Moshfeghi Andrew A, Puliafito Carmen A (2005). Optical coherence tomography findings after an intravitreal injection of bevacizumab (Avastin^®^) for neovascular age-related macular degeneration. Ophthalmic Surg Lasers Imaging Retina.

[16] Iturralde D (2006). Intravitreal bevacizumab (Avastin) treatment of macular edema in central retinal vein occlusion: a short-term study. Retina.

[17] Costa RA (2007). Intravitreal bevacizumab (avastin) for central and hemicentral retinal vein occlusions: IBeVO study. Retina.

[18] Regatieri CV, Branchini L, Carmody J, Fujimoto JG, Duker JS (2012). Chorodial thickness in patients with diabetic retinopathy analyzed by spectral-domain optical coherence tomography. Retina.

[19] Unsal E, Eltutar K, Zirtiloglu S, Dincer N, Ozdogan Erkul S, Gungel H (2014). Choroidal thickness in patients with diabetic retinopathy. Clin Ophthalmol.

[20] Shao L (2014). Visual acuity and subfoveal choroidal thickness: the Beijing Eye Study. Am J Ophthalmol.

[21] Nourinia R (2018). Changes in central choroidal thickness after treatment of diabetic macular edema with intravitreal bevacizumab correlation with central macular thickness and best-corrected visual acuity. Retina.

[22] Ota M, Tsujikawa A, Kita M, Miyamoto K, Sakamoto A, Yamaike N (2008). Integrity of foveal photoreceptor layer in central retinal vein occlusion. Retina.

[23] Heier JS, Campochiaro PA, Yau L, Li Z, Saroj N, Rubio RG (2012). Ranibizumab for macular edema due to retinal vein occlusions: long-term follow-up in the HORIZON trial. Ophthalmology.

[24] Central Vein Occlusion Study Group (1995). Evaluation of grid pattern photocoagulation for macular edema in central vein occlusion. The central vein occlusion study group M report. Ophthalmology.

